# ImageNet, RadiologyNET, or RadImageNet? Evaluating Pretraining Across CT, MRI, and X-Ray Classification

**DOI:** 10.3390/jimaging12100501

**Published:** 2026-10-10

**Authors:** Luka Kršul, Dominik Vičević, Franko Hržić, Ivan Štajduhar, Mateja Napravnik

**Affiliations:** 1 Faculty of Engineering, University of Rijeka, 51000 Rijeka, Croatiadominik.vicevic@uniri.hr (D.V.); franko.hrzic@uniri.hr (F.H.); ivan.stajduhar@uniri.hr (I.Š.); 2 Center for Artificial Intelligence and Cybersecurity, 51000 Rijeka, Croatia; 3 Department of Polytechnics, Dr. Franjo Tuđman Defense and Security University, 10000 Zagreb, Croatia

**Keywords:** transfer learning, medical image classification, convolutional neural networks, ImageNet, RadiologyNET, RadImageNet

## Abstract

Due to the limited availability of annotated medical datasets, medical image classification typically relies on transfer learning from large-scale natural image datasets such as ImageNet. However, it remains unclear when domain-specific pretraining offers greater benefits. This study evaluates the impact of different pretrained weights on neural network performance in radiological image classification tasks. Four strategies were compared: ImageNet, RadiologyNET, and RadImageNet model fine-tuning against learning from randomly initialised weights (Baseline). Experiments were conducted over ten seed-paired runs using ResNet50, InceptionV3, DenseNet121, EfficientNet-B3, and MobileNetV3-Large on three classification tasks: multiclass Chest CT, multiclass BRISC brain MRI, and binary FracAtlas X-ray fracture classification. Pretraining generally improved performance over the Baseline, with the largest improvements observed on the Chest CT task. The Friedman tests identified significant differences between initialisation strategies for every architecture on all three tasks (all p≤0.0002). ImageNet achieved the best results in most architecture–task combinations, while RadiologyNET was comparable or superior in selected cases, including three of five architectures on Chest CT. RadImageNet showed less consistent transfer, particularly with DenseNet121. Pretrained models also generally reached high validation performance more rapidly than randomly initialised models. These results further support previous findings that ImageNet-pretrained models can perform comparably to or better than domain-specific radiological models when fine-tuned on sufficient downstream data, while the observed differences are consistent with possible effects of source–target alignment, downstream data availability, and network architecture.

## 1. Introduction

Worldwide, more than 4.2 billion medical radiological examinations are performed annually [1]. The English National Health Service recorded approximately 23.5 million plain-radiography examinations, 11.5 million ultrasound, 8.3 million computed tomography (CT), and 4.8 million magnetic resonance imaging (MRI) examinations in 2024/25 [2]. Plain radiography and CT together accounted for approximately 64% of recorded imaging activity. However, high acquisition volume does not directly translate into task-ready datasets. Although self-supervised and semi-supervised methods can learn useful representations from large collections of unlabelled images [3], clinically reliable annotations remain expensive, expert-dependent, and limited by privacy requirements [4,5]. The conventional transfer learning approach in medical imaging is to initialise a network with weights pretrained on a large natural-image dataset, most commonly ImageNet [6], and then fine-tune it for the target radiological task [7,8,9]. This strategy can improve optimisation and reduce overfitting when labelled target data are limited.

Several studies have investigated whether pretraining on medical images provides a more effective alternative to ImageNet. RadImageNet [10] was introduced as a large-scale radiological dataset containing CT, MRI, and ultrasound images from a wide range of anatomical regions and pathologies. Models pretrained on RadImageNet were reported to outperform ImageNet-pretrained models across eight downstream classification and segmentation tasks spanning several modalities (MRI, CT, ultrasound, radiography), with noticeable improvements on smaller datasets [10]. Other studies have also shown that domain-specific or self-supervised pretraining can be advantageous when the source and target modalities are closely matched. For example, CT-specific pretraining has been shown to improve transfer to CT-based tasks, while its benefits may not extend consistently to other radiology imaging modalities [11]. However, the superiority of domain-specific pretraining is not consistent across studies. A replication of the RadImageNet evaluation found that ImageNet-pretrained models outperformed RadImageNet models on most of the investigated tasks, with results being sensitive to hyperparameters, dataset composition, and fine-tuning strategy [12]. In our previous work, a large radiological dataset, RadiologyNET [13] (consisting of CT, MRI, radiofluoroscopy, X-ray angiography and computed radiography), was used to pretrain several neural network architectures and evaluated against ImageNet-pretrained and randomly initialised models across several medical imaging tasks. The results indicated that RadiologyNET weights offered occasional advantages in resource-limited or closely aligned domains but did not consistently outperform ImageNet [14]. Furthermore, it was shown that some transfer learning configurations may effectively hinder downstream performance, as a reconstruction-pretrained U-Net significantly underperformed compared to a randomly initialised U-Net [14]. Taken together, these findings suggest that the value of medical-domain pretraining depends not only on the size of the pretraining dataset [15], but also on domain alignment, task type, downstream dataset size, model architecture, and the fine-tuning process.

**Figure 1:**
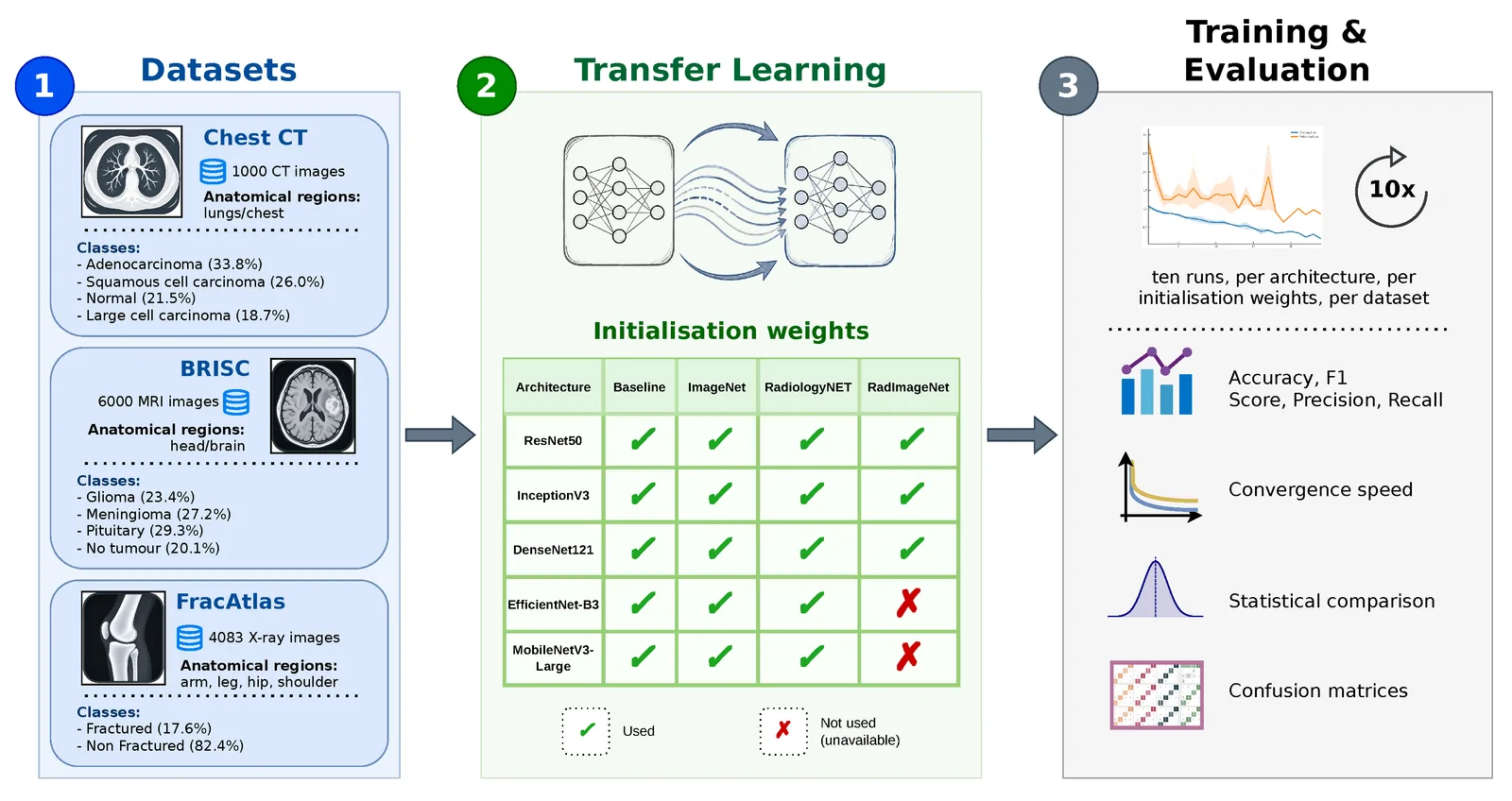
Overview of the experimental workflow. Chest CT, BRISC and FracAtlas image datasets were used to evaluate five neural network architectures initialised with ImageNet, RadiologyNET, RadImageNet, or random (Baseline) weights. Each architecture–initialisation combination was trained in ten runs with paired seeds and compared statistically.

In this study, we build on our previous evaluation of RadiologyNET foundation models and extend the comparison to include RadImageNet-pretrained models. Specifically, we compare four initialisation strategies: (i) random weights (Baseline), (ii) ImageNet-pretrained weights, (iii) RadiologyNET-pretrained weights, and (iv) RadImageNet-pretrained weights. The evaluation is performed across three classification tasks covering different imaging modalities: multiclass Chest CT classification [16], multiclass brain tumour MRI classification (BRain tumor Image Segmentation and Classification, BRISC [17]), and binary X-ray fracture classification (FracAtlas [18]). For this purpose, multiple well-established neural network architectures were evaluated, including ResNet50 [19], InceptionV3 [20], EfficientNet-B3 [21], MobileNetV3-Large [22], and DenseNet121 [23]. To the best of our knowledge, this is the first study comparing the effectiveness of transfer learning from ImageNet, RadiologyNET, and RadImageNet weights. The conducted experiment is illustrated in Figure 1.

## 2. Materials and Methods

### 2.1. Datasets

This study employed multiple publicly available medical imaging datasets, selected to differ in size, image modality, and classification task. A summary is provided in Figure 1. To prevent possible data leakage, every dataset was checked for duplicate images by comparing their SHA-256 hashes, and any identified duplicates were either removed or assigned to a single subset. For all three datasets, images were resized to 300×300 pixels and normalised prior to training. After this, every dataset was preprocessed as described below.

The Multiclass Chest CT Scan dataset [16] comprises CT images used for the classification of non-small cell lung cancer (NSCLC) subtypes. It contains a total of 1000 images distributed across four classes: normal (no finding), adenocarcinoma, large cell carcinoma, and squamous cell carcinoma. After removing duplicate images, the remaining 944 images were split into training, validation, and test subsets in an approximately 70/10/20 ratio.

To include MRI data in our experiments, the BRISC 2025 dataset [17] was used, providing 6000 T1-weighted MRI slices across four classes (glioma, meningioma, pituitary and no tumour). As the official release does not provide a validation split, the full dataset was partitioned into 4500 training, 500 validation, and 1000 test images. After resizing to 300×300 pixels, nine test images became byte-identical to training images, as adjacent MRI slices are highly similar and the reduced resolution removed the remaining differences between them. To prevent leakage, these duplicates were moved to the training set, resulting in a final split of 4509 training, 500 validation, and 991 test images.

The FracAtlas dataset [18] consists of 4083 X-ray images of the arm, leg, hip, and shoulder, divided into two classes: fractured (717 images) and non-fractured (3366 images). The dataset was split into stratified training, validation, and test sets, after which the fractured class was oversampled within the training split to match the number of images in the non-fractured class. Oversampling was performed by resampling fractured images with replacement and applying random augmentations (vertical flip, rotation, affine transformation, and brightness/contrast jitter), which resulted in 4712 images in training, 613 in validation and 612 in the test split.

### 2.2. Pretrained Weights

Models initialised with random weights (the default torchvision initialisation, i.e., Kaiming/He initialisation [24] for all architectures except InceptionV3, which uses a truncated normal distribution) were used as the reference and are denoted as Baseline. The other configurations used ImageNet-, RadiologyNET-, or RadImageNet-pretrained weights, with the corresponding pretraining datasets summarised in Table 1. While ImageNet consists of a large collection of high-resolution natural images, RadiologyNET and RadImageNet are composed of radiological images and are expected to provide feature representations more closely aligned with the medical domain. RadImageNet weights were pretrained on a large dataset (1.3 million images) comprising three radiology imaging modalities: MRI (49.6%), ultrasound (28.8%), and CT (21.6%). RadiologyNET was pretrained on 1.9 million images consisting of five modalities dominated by CT (53.7%), followed by MRI (22.7%), X-ray angiography (19.6%), computed radiography (2.7%), and radiofluoroscopy (1.3%). RadImageNet was organised into 165 pathological labels based on clinical interpretations [10], while RadiologyNET was pseudo-labelled through an unsupervised pipeline into 36 semantically similar groups [13]. Both datasets were previously [10,14] used to pretrain several commonly used neural network architectures through supervised learning.

### 2.3. Experiment Setup

The following neural network architectures were evaluated in this study: ResNet50, InceptionV3, EfficientNet-B3, MobileNetV3-Large, and DenseNet121. These models were selected due to their well-established use in image classification tasks and the availability of pretrained weights from multiple sources. In all scenarios, the networks were fully fine-tuned on the target datasets without freezing any layers. Apart from the initial weights, the only difference was the classification head of the RadImageNet models, which followed the original RadImageNet implementation instead of the standard torchvision head. Because of limited weight availability, RadImageNet initialisation was evaluated only for ResNet50, InceptionV3, and DenseNet121.

All models were trained under identical conditions to ensure a fair comparison. Images were normalised with ImageNet channel statistics for all initialisation strategies, and no online augmentation was applied. Training was performed for a minimum of 10 and a maximum of 100 epochs with a batch size of 32. Early stopping was applied based on validation loss to prevent overfitting, terminating training when the validation loss did not decrease by at least $10^{-4}$ for 5 consecutive epochs. The Adam optimiser [25] was used with a fixed learning rate of $1\times 10^{-4}$, and the cross-entropy loss function was applied for all tasks. The model parameters with the lowest validation loss were saved and used for final evaluation on the test set.

Each experiment was repeated across ten runs using the same fixed training, validation, and test sets, where the *i*-th run of every combination of architecture, weight initialisation, and dataset used the same random seed. The hardware configuration used for all experiments is described in Appendix A. Model performance was evaluated using standard classification metrics: accuracy, precision, recall, and F1-score, computed on both the validation and test sets. For the multiclass Chest CT and BRISC tasks, all metrics (including accuracy) were macro-averaged. Final test results are reported as the mean and standard deviation of these metrics across the ten runs. To determine whether the differences between initialisation strategies were statistically significant, a Friedman test [26] was conducted separately for each architecture and dataset, comparing the F1-scores of the initialisation strategies between runs that shared the same random seed, with a significance level of α=0.05. Where the Friedman test indicated a potentially significant difference, post hoc pairwise comparisons were performed using the Wilcoxon signed-rank test [27] on the seed-paired F1-scores, with Holm correction for multiple comparisons [28], following established practice for the comparison of classifiers across repeated experimental conditions [29]. To visualise how pretraining affected the learned feature space, t-SNE [30] was applied to the penultimate-layer representations, following similar use in medical representation-learning studies [31,32]. However, because t-SNE preserves local neighbourhoods and can distort global distances [30], these projections should be interpreted only as supporting evidence for the quantitative results.

## 3. Results

**Figure 2:**
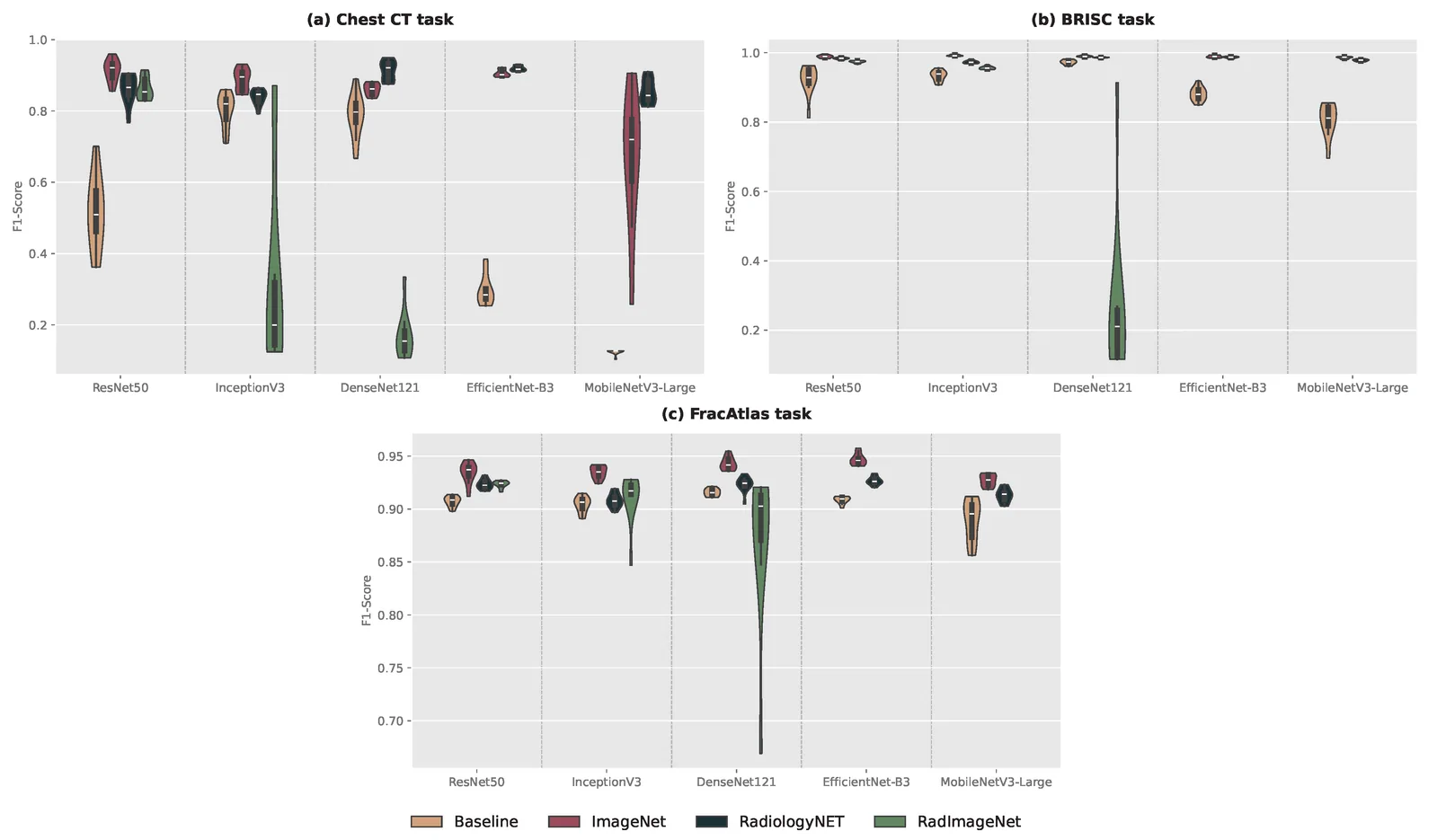
Distribution of test F1-scores across ten runs for all models and weight initialisation strategies: (**a**) Chest CT task, (**b**) BRISC task. (**c**) FracAtlas task.

**Figure 3:**
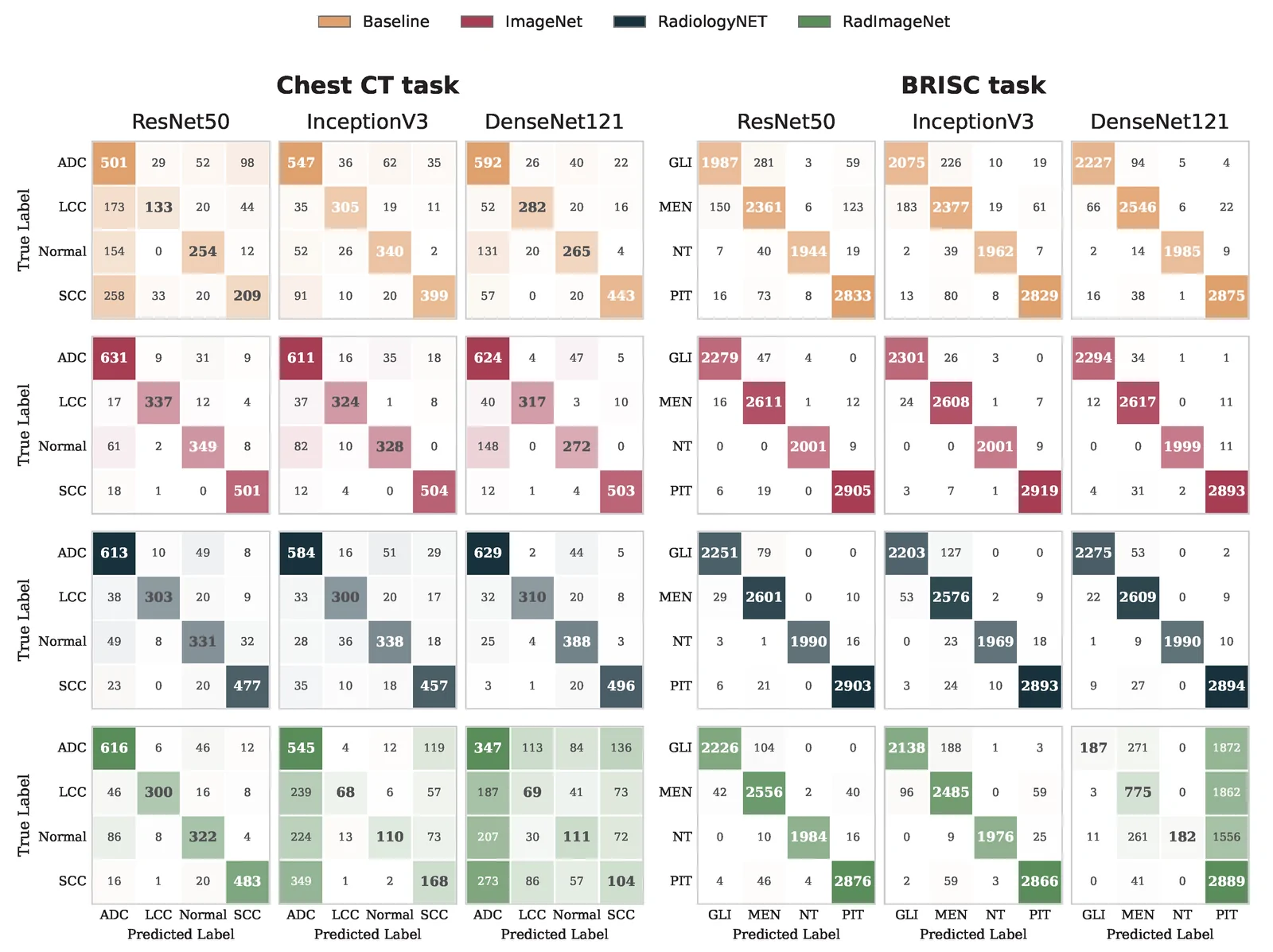
Test-set confusion matrices for the Chest CT (**left**) and BRISC brain MRI (**right**) tasks for ResNet50, InceptionV3, and DenseNet121. Rows correspond to the initialisation strategy (Baseline, ImageNet, RadiologyNET, and RadImageNet). Each matrix sums the predictions of all ten runs. Numbers given in bold indicate correct predictions; darker shades correspond to higher prediction counts. *ADC—Adenocarcinoma*, *LCC—Large cell carcinoma*, *SCC—Squamous cell carcinoma*, *GLI—Glioma*, *MEN—Meningioma*, *NT—no tumour*, *PIT—Pituitary tumour*.

Table 2, Table 3 and Table 4 report the mean and standard deviation of accuracy, precision, recall, and F1-score across ten runs for the Chest CT scan, BRISC, and FracAtlas datasets, respectively. Figure 2 shows the corresponding distributions of test F1-scores across runs for all datasets. Table 5 reports the Friedman test statistics and corresponding degrees of freedom and *p*-values for each architecture and dataset, together with the Holm-corrected *p*-values from the post hoc pairwise Wilcoxon signed-rank tests comparing each pair of initialisation strategies. Figure 3 shows the confusion matrices for the Chest CT and BRISC tasks, aggregated over all runs, for the three architectures evaluated under all four initialisation strategies.

**Figure 4:**
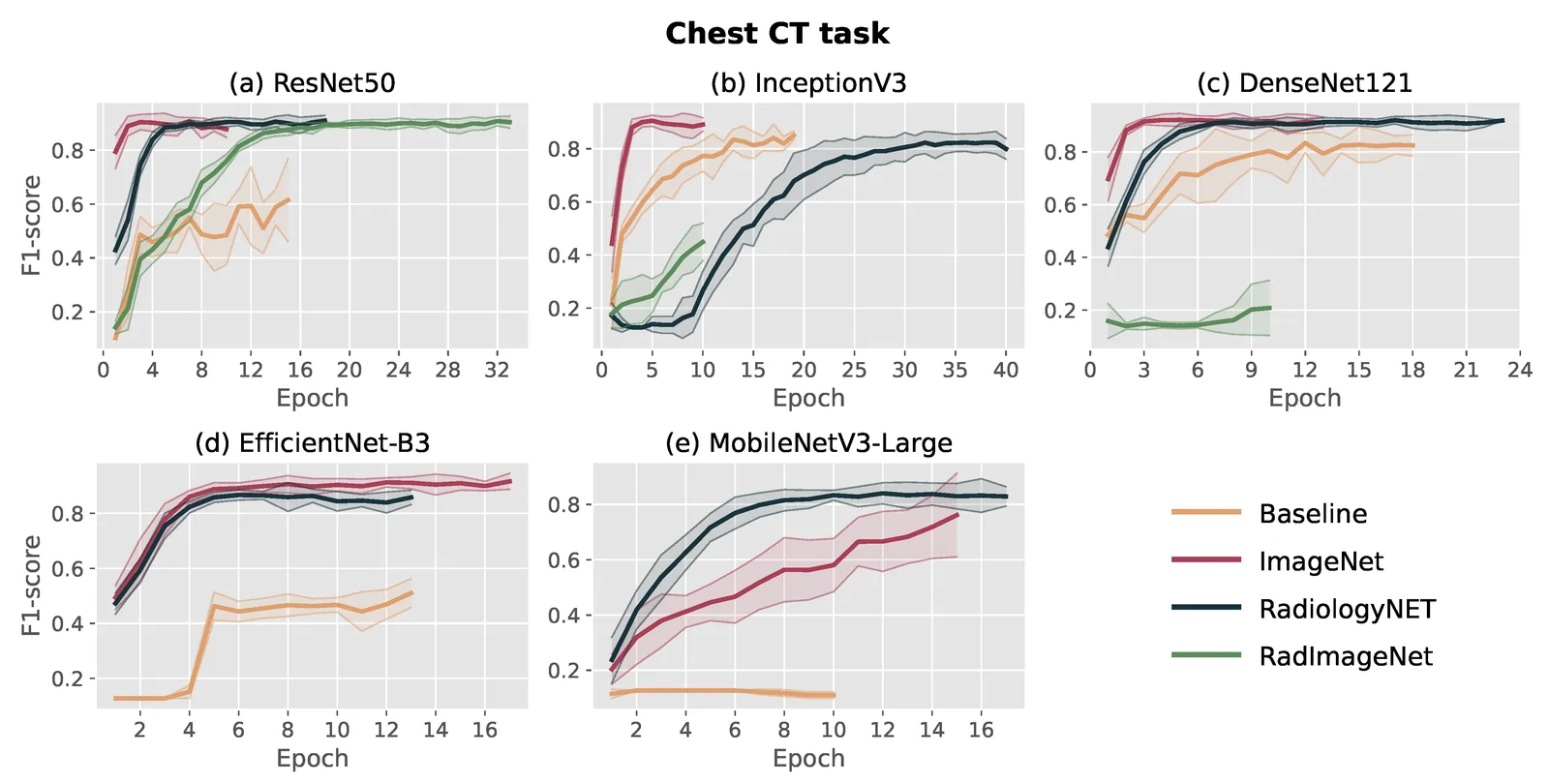
Average validation F1-score across training epochs for all tested architectures under all weight-initialisation strategies for the Chest CT dataset. Solid lines represent the mean across repeated runs, and shaded regions indicate standard deviation. (**a**) ResNet50, (**b**) InceptionV3, (**c**) DenseNet121, (**d**) EfficientNet-B3, (**e**) MobileNetV3-Large.

**Figure 5:**
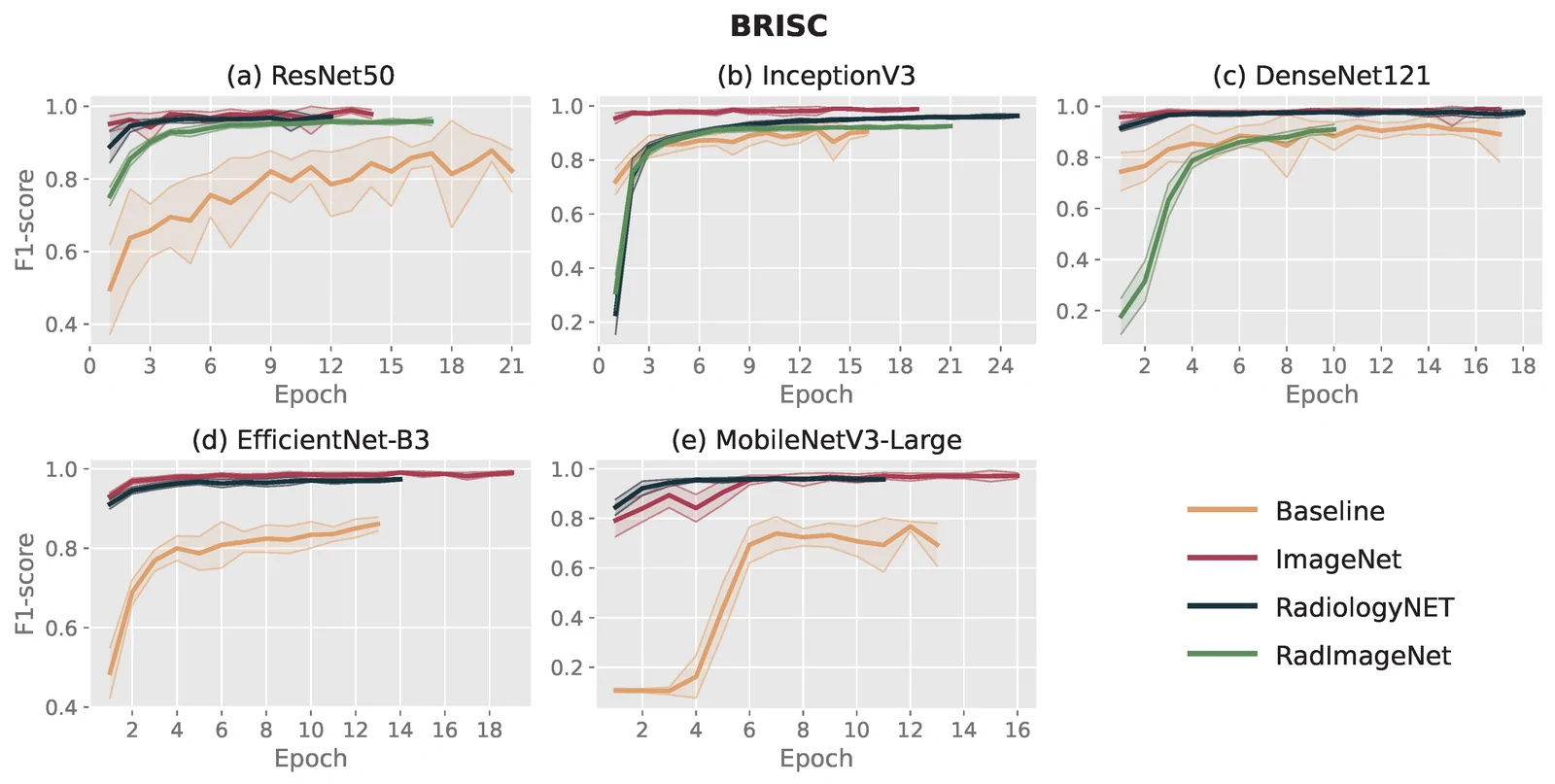
Average validation F1-score across training epochs for all tested architectures under all weight-initialisation strategies for the BRISC dataset. Solid lines represent the mean across repeated runs, and shaded regions indicate standard deviation. (**a**) ResNet50, (**b**) InceptionV3, (**c**) DenseNet121, (**d**) EfficientNet-B3, (**e**) MobileNetV3-Large.

**Figure 6:**
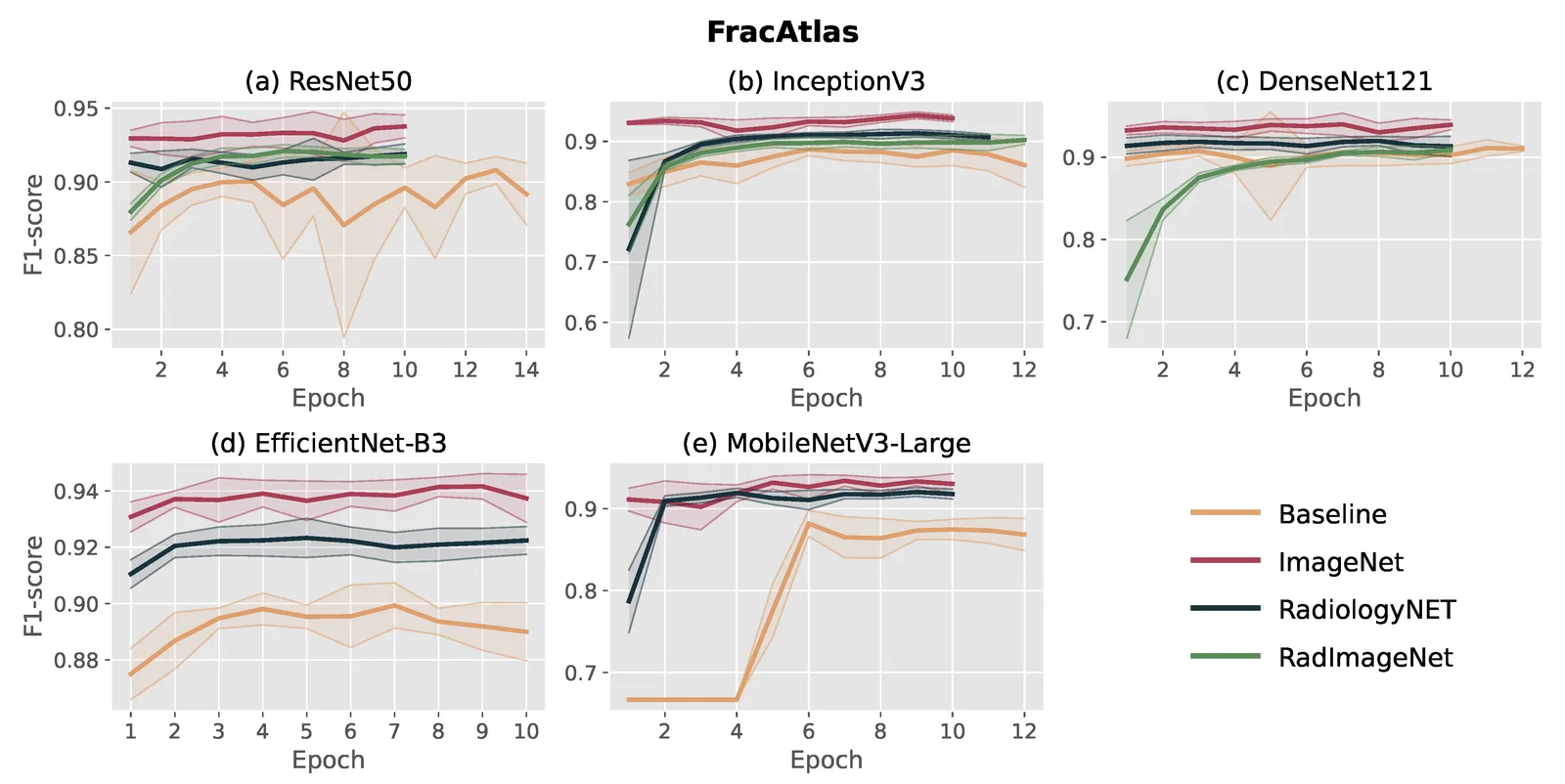
Average validation F1-score across training epochs for all tested architectures under all weight-initialisation strategies for the FracAtlas dataset. Solid lines represent the mean across repeated runs, and shaded regions indicate standard deviation. (**a**) ResNet50, (**b**) InceptionV3, (**c**) DenseNet121, (**d**) EfficientNet-B3, (**e**) MobileNetV3-Large.

**Figure A1:**
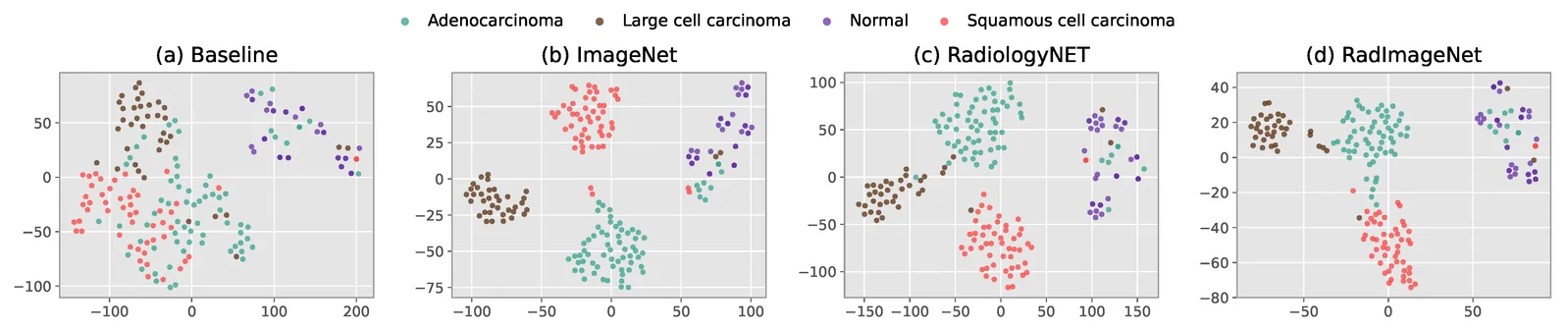
t-SNE visualisation of feature embeddings extracted from a ResNet50 model on the Chest CT scan dataset, initialised with different weight strategies: (**a**) Baseline. (**b**) ImageNet. (**c**) RadiologyNET. (**d**) RadImageNet.

**Figure A2:**
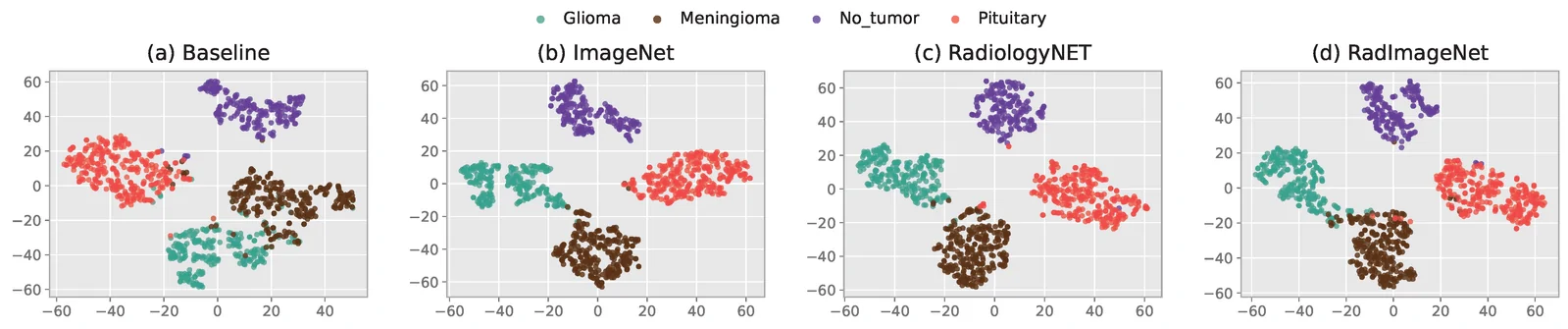
t-SNE visualisation of feature embeddings extracted from a ResNet50 model on the BRISC dataset, initialised with different weight strategies: (**a**) Baseline. (**b**) ImageNet. (**c**) RadiologyNET. (**d**) RadImageNet.

**Figure A3:**
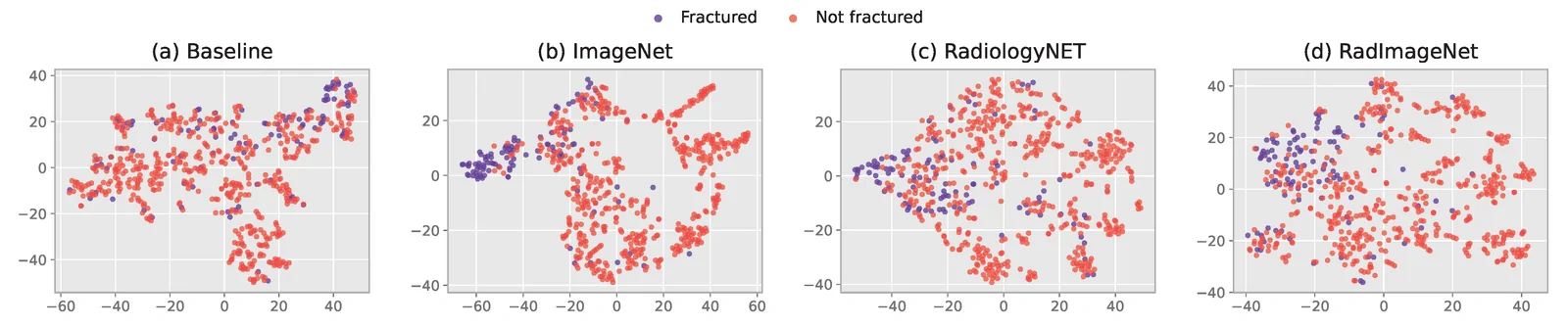
t-SNE visualisation of feature embeddings extracted from a ResNet50 model on the FracAtlas dataset, initialised with different weight strategies: (**a**) Baseline. (**b**) ImageNet. (**c**) RadiologyNET. (**d**) RadImageNet.

Figure 4 shows the mean validation F1-score per epoch for the Chest CT dataset, with shaded regions indicating the standard deviation across runs. Each curve is shown up to the last epoch reached by at least half of the runs for the corresponding configuration. The same can be seen in Figure 5 and Figure 6 for the BRISC and FracAtlas datasets, respectively. Table A1 in Appendix A reports the average training time per run for each architecture and dataset. For every architecture, training was shortest on the Chest CT scan dataset and longest on the BRISC dataset. Across all three datasets, MobileNetV3-Large was the fastest architecture to train, with an overall mean of 235.1 s per run, whereas DenseNet121 was the slowest, with 586.0 s per run. Additionally, we provide the t-SNE projections for all datasets in Appendix B, Figure A1, Figure A2 and Figure A3.

### 3.1. Chest CT Scan Dataset

On the Chest CT scan dataset, pretrained models generally achieved higher test performance compared to randomly initialised models (Table 2). ImageNet initialisation produced the highest mean F1-score for ResNet50 and InceptionV3 (0.9126 and 0.8873, respectively), whereas RadiologyNET performed best for DenseNet121, EfficientNet-B3, and MobileNetV3-Large (0.9127, 0.9190, and 0.8548, respectively). The Baseline configuration produced the lowest mean F1-score for ResNet50, EfficientNet-B3, and MobileNetV3-Large, with the largest difference observed for MobileNetV3-Large, where Baseline remained at an F1-score of 0.1250 with an accuracy of 0.25. For InceptionV3 and DenseNet121, RadImageNet produced the lowest mean F1-scores (0.3284 and 0.1700). The distributions shown in Figure 2 additionally indicate greater run-to-run variability for several configurations, particularly ImageNet with MobileNetV3-Large and RadImageNet with InceptionV3.

The Friedman test indicated statistically significant differences between initialisation strategies for every architecture on the Chest CT dataset (Table 5). Post hoc testing showed that ImageNet significantly outperformed Baseline for all five architectures, and RadiologyNET did so for all architectures except InceptionV3, where the difference was not significant. RadImageNet significantly outperformed Baseline for ResNet50, whereas it performed significantly worse than Baseline for InceptionV3 and DenseNet121. ImageNet and RadiologyNET differed significantly for all five architectures, with ImageNet performing better for ResNet50 and InceptionV3, and RadiologyNET performing better for DenseNet121, EfficientNet-B3, and MobileNetV3-Large. RadiologyNET significantly outperformed RadImageNet for InceptionV3 and DenseNet121, while no significant difference was observed between them for ResNet50.

The confusion matrices in Figure 3 show that, for the ImageNet and RadiologyNET configurations, most misclassifications occurred between adenocarcinoma and the normal class and, to a lesser extent, between large cell carcinoma and adenocarcinoma. The Baseline ResNet50 frequently predicted adenocarcinoma for samples of the other carcinoma subtypes. The RadImageNet-initialised InceptionV3 predicted mostly adenocarcinoma or squamous cell carcinoma regardless of the true class, whereas the predictions of the RadImageNet-initialised DenseNet121 were distributed across all classes with little relation to the true label.

The validation trajectories in Figure 4 show that ImageNet-initialised models generally reached high validation F1-scores within relatively few epochs, with the exception of MobileNetV3-Large, which improved more gradually. RadiologyNET also converged rapidly for ResNet50, EfficientNet-B3, MobileNetV3-Large, and DenseNet121, while convergence was slower for InceptionV3. RadImageNet converged more slowly for ResNet50 and InceptionV3, and remained at a validation F1-score of approximately 0.2 for DenseNet121. Baseline configurations generally improved more slowly and reached lower validation performance, with MobileNetV3-Large failing to develop a competitive solution during training.

### 3.2. BRISC Dataset

On the BRISC dataset, nearly all pretrained configurations achieved mean F1-scores above 0.95 (Table 3). ImageNet initialisation produced the highest mean F1-score for all five architectures, reaching 0.9888, 0.9919, 0.9896, 0.9889, and 0.9857 for ResNet50, InceptionV3, DenseNet121, EfficientNet-B3, and MobileNetV3-Large, respectively. RadiologyNET ranked second for every architecture, with mean F1-scores ranging from 0.9729 (InceptionV3) to 0.9866 (EfficientNet-B3). RadImageNet performed worse than both ImageNet and RadiologyNET wherever corresponding weights were available, reaching 0.9738 for ResNet50 and 0.9562 for InceptionV3, while DenseNet121 failed in nine of ten runs, resulting in a mean F1-score of 0.2597 with a standard deviation of 0.2264. The Baseline configuration produced the lowest mean F1-score for all architectures except DenseNet121, with the largest difference observed for MobileNetV3-Large (0.8054). This is also reflected in the F1-score distributions in Figure 2.

The Friedman test indicated statistically significant differences between initialisation strategies for every architecture on the BRISC dataset (Table 5). Post hoc testing showed that all pairwise differences were statistically significant, with the sole exception of ImageNet and RadiologyNET for EfficientNet-B3. Notably, for DenseNet121, Baseline significantly outperformed RadImageNet.

The confusion matrices in Figure 3 show that, for the ImageNet and RadiologyNET configurations, the *no tumour* samples were classified almost without error.

Most misclassifications occurred between glioma and meningioma and were most frequent for Baseline, particularly for ResNet50. For DenseNet121 initialised with RadImageNet weights, the majority of samples from every class were predicted as pituitary tumour.

The validation trajectories in Figure 5 show that ImageNet-initialised models reached validation F1-scores above 0.95 from the first epochs for all architectures. RadiologyNET converged similarly rapidly, requiring only a few additional epochs for InceptionV3. RadImageNet converged within a few epochs for ResNet50 and InceptionV3, but more slowly for DenseNet121, where its validation F1-score increased over the first nine epochs before approaching that of the remaining pretrained strategies. The Baseline configurations reached lower and more variable validation F1-scores, particularly for ResNet50, while the Baseline MobileNetV3-Large remained at a validation F1-score of approximately 0.1 for the first four epochs before improving.

### 3.3. FracAtlas Dataset

The performance on the FracAtlas dataset was lower overall than on the BRISC dataset, with most configurations achieving mean F1-scores between 0.87 and 0.95 (Table 4). ImageNet achieved the highest mean F1-score for all five architectures: ResNet50 (0.9342), InceptionV3 (0.9347), DenseNet121 (0.9429), EfficientNet-B3 (0.9467), and MobileNetV3-Large (0.9272). RadImageNet performed similarly to RadiologyNET for ResNet50 and InceptionV3, but produced the lowest mean performance for DenseNet121, where its mean F1-score was 0.8729 with a standard deviation of 0.0716. This larger variability, also present to a lesser extent for RadImageNet with InceptionV3, is visible in the F1-score distributions in Figure 2.

Significant differences between initialisation strategies were again identified by the Friedman test for every architecture (Table 5). For ResNet50, ImageNet significantly outperformed Baseline and RadImageNet, while its difference from RadiologyNET was not statistically significant (p=0.0547); no significant difference was observed between RadiologyNET and RadImageNet. For InceptionV3, ImageNet significantly outperformed all other strategies, whereas no significant differences were detected among Baseline, RadiologyNET, and RadImageNet. For DenseNet121, all pairwise differences were statistically significant. For EfficientNet-B3 and MobileNetV3-Large, all three available initialisation strategies differed significantly, with ImageNet achieving the highest mean F1-score and Baseline the lowest.

The validation curves in Figure 6 show that ImageNet-initialised models generally achieved the highest validation F1-scores from the first epoch onwards, followed by RadiologyNET, which reached its plateau within the first few epochs. RadImageNet started from lower validation F1-scores and converged towards the level of RadiologyNET, most slowly for DenseNet121. The Baseline configurations typically reached lower and more variable validation F1-scores, with the Baseline MobileNetV3-Large remaining at approximately 0.67 for the first four epochs before improving. Despite these differences in validation trajectories, the final test performance of the ImageNet and RadiologyNET configurations differed by only a few percentage points.

## 4. Discussion

Across all three tasks, pretraining generally showed an advantage over random initialisation, although the extent of this advantage depended on the downstream task, particularly on the amount of available downstream data and its overlap with the pretraining domain. Pretrained models generally reached higher validation performance more rapidly than Baseline (Figure 4, Figure 5 and Figure 6), whereas the best-performing pretraining source varied between tasks and architectures.

***Relationship with downstream data size.*** The benefit of pretraining was most noticeable on Chest CT, which was the most challenging task in the current experimental setting: it contained relatively few samples for a four-class problem, produced the lowest overall F1-scores, and showed the largest differences between initialisation strategies (Table 2). Several Baseline models failed to converge (e.g., MobileNetV3-Large), and the corresponding confusion matrices (Figure 3) and t-SNE projections (Figure A1) show overlap among the carcinoma subtypes for Baseline. In contrast, BRISC and FracAtlas contained more training data, and Baseline did not fail on either task, although it remained significantly below the best pretrained strategy for every architecture (Table 3, Table 4 and Table 5). This supports previous findings that transfer learning is most beneficial when downstream data alone are insufficient for stable learning [14,15].

***Relationship with source-target alignment.*** ImageNet achieved the best results in most architecture–task combinations, while RadiologyNET was superior for three of the five architectures on Chest CT. Chest CT was the task with both the smallest training set and the largest modality overlap with RadiologyNET (53.7% CT images), which also significantly outperformed RadImageNet on this task for InceptionV3 and DenseNet121. On BRISC, RadiologyNET remained within one percentage point of ImageNet for all architectures except InceptionV3; although most of these differences were statistically significant, their practical difference is small (≤0.0052 difference in F1-score for ResNet50 and DenseNet121). For MobileNetV3-Large, RadiologyNET also provided a better start to training (Figure 5e), which may reflect its MRI content, but ImageNet matched it within a few epochs, suggesting that the larger training set was sufficient for ImageNet features to adapt. In contrast, despite its large proportion of MRI data [10], RadImageNet was the weakest pretrained strategy on BRISC for every available architecture, which could be attributed to its MRI data spanning many anatomical regions (head examinations are better represented in RadiologyNET [13]). On FracAtlas, where neither radiological source contains substantial radiograph data, ImageNet achieved the highest mean F1-score for all architectures and outperformed RadiologyNET significantly for four of the five architectures; the ResNet50 comparison did not reach statistical significance (p=0.0547). RadiologyNET and RadImageNet performed similarly for ResNet50 and InceptionV3, whereas RadiologyNET performed significantly better with DenseNet121. The successful transfer to chest radiographs reported for RadImageNet [10] did not consistently extend to limb fractures in this study. This is consistent with our previous findings that source-domain alignment can benefit transfer learning, particularly when pretraining and downstream data overlap, but that more diverse pretraining data may yield more generalisable representations [14].

***Relationship with network architecture.*** The results also indicate an interaction between pretraining strategy and architecture, as no single initialisation was optimal across all backbones. This was most evident on Chest CT: ImageNet performed better with larger ResNet50 and InceptionV3, whereas RadiologyNET performed best with DenseNet121, EfficientNet-B3, and MobileNetV3-Large, where the observed difference was most noticeable. One possible explanation is that, as the smallest model in this experiment, MobileNetV3-Large may have less capacity to adapt generic ImageNet features during fine-tuning and therefore benefits more from representations already aligned with the target domain. This trend is visible in our previous work [14] and is consistent with evidence that the advantage of ImageNet pretraining over random initialisation decreases as backbone capacity is reduced [33]. As BRISC and FracAtlas contained more training data, this effect was less visible, but Figure 5e indicates that RadiologyNET boosted early performance on the BRISC dataset in MobileNetV3-Large. A separate architecture-specific effect was observed for RadImageNet-initialised DenseNet121, which underperformed across all three tasks. While observing Figure 3, it can be seen that DenseNet121 performed normally with ImageNet and RadiologyNET, so this behaviour may reflect sensitivity to the pretrained checkpoint or fine-tuning rather than source-target mismatch or network learning capacity.

***Stability and convergence between runs.*** Random initialisation can make training more sensitive to the starting parameter configuration, while pretrained models have a consistent starting point. Consequently, the latter showed more stable validation trajectories and reached high F1-scores more rapidly, with some exceptions (e.g., RadiologyNET and RadImageNet in Figure 4b and ImageNet in Figure 4e). Baseline models either showed greater run-to-run variability or, in some cases, consistently converged to poor solutions (e.g., MobileNetV3-Large on the Chest CT task). Several RadImageNet configurations also showed slower or less stable convergence, particularly DenseNet121 (Figure 4c and Figure 6c). These differences in convergence were also reflected in training time (Table A1), although shorter training did not necessarily indicate better optimisation because early stopping could terminate poorly performing configurations earlier.

***Limitations.*** First, all initialisation strategies were evaluated using the same optimisation settings; however, the optimal fine-tuning configuration may differ between pretrained sources (e.g., for RadImageNet-initialised DenseNet121). Second, this study uses conventional, well-established convolutional neural network architectures and did not benchmark performance on newer transformer-based [34] networks, due to the (un)availability of pretrained weights from RadImageNet. Third, downstream dataset size, source-target overlap, and network capacity were not independently manipulated in the present experimental design; therefore, the observed associations should be interpreted as post hoc hypotheses rather than evidence of causal mechanisms. Finally, although the three downstream datasets include CT, MRI, and radiography, they cover a limited range of possible domain overlap. Future work should further expand the experimental evaluation with different source-target alignment, for example, with ultrasound data, which are not present in RadiologyNET.

## 5. Conclusions

This study extends our previous comparison of ImageNet, RadiologyNET, and random initialisation by introducing RadImageNet and conducting a ten-run evaluation across five neural network architectures and three radiological classification tasks [14]. The results align with our previous findings that transfer learning is particularly beneficial when downstream data are limited, while its effectiveness varied across tasks and architectures. The observed patterns are consistent with possible roles for source–target alignment and downstream data availability, although these factors were not independently manipulated in the present study. ImageNet achieved the strongest results in most architecture-task combinations, while RadiologyNET was competitive or superior when the pretraining and target domains were more closely aligned and less training data were available (i.e., the Chest CT task).

Pretrained weights should therefore be selected by considering the composition and biases of the source dataset, its modality and anatomical overlap with the target task, the downstream data available, and the network architecture. The Chest CT results suggest that domain-aligned pretraining may be particularly useful for lower-capacity networks when downstream data are limited. Future work should evaluate these interactions across additional source-target combinations and fine-tuning settings, particularly on ultrasound, which is represented in RadImageNet but absent from RadiologyNET, as well as on further MRI tasks with differing anatomical overlap. Such experiments could further clarify how source-data diversity and source-target alignment relate to the transferability of learned features.

## Figures and Tables

**Table 1:** Comparison of the pretraining datasets used in this study. CT: computed tomography; MRI: magnetic resonance imaging; US: ultrasound; XA: X-ray angiography; CR: computed radiography; RF: radiofluoroscopy; “–”: not applicable (ImageNet contains natural RGB images only, with no medical imaging modalities).

Dataset	Images	Labels	Modality Composition (%)					
			CT	MRI	US	XA	CR	RF
ImageNet	1.28 M	1000 classes	–	–	–	–	–	–
			*(natural RGB images)*					
RadiologyNET	1.9 M	36 pseudo-labels	53.7	22.7	–	19.6	2.7	1.3
RadImageNet	1.3 M	165 pathological labels	21.6	49.6	28.8	–	–	–

**Table 2:** Results on the Chest CT scan dataset, reported as mean ± standard deviation across ten runs. The highest value per metric within each architecture is shown in bold.

Model	Weight	Accuracy	Precision	Recall	F1
ResNet50	Baseline	0.5257 ± 0.0865	0.6202 ± 0.0645	0.5257 ± 0.0865	0.5160 ± 0.0980
	ImageNet	**0.9083** ± **0.0358**	**0.9270** ± **0.0176**	**0.9083** ± **0.0358**	**0.9126** ± **0.0293**
	RadiologyNET	0.8564 ± 0.0431	0.8769 ± 0.0342	0.8564 ± 0.0431	0.8619 ± 0.0408
	RadImageNet	0.8531 ± 0.0310	0.8784 ± 0.0275	0.8531 ± 0.0310	0.8624 ± 0.0295
InceptionV3	Baseline	0.8014 ± 0.0394	0.8142 ± 0.0460	0.8014 ± 0.0394	0.8016 ± 0.0453
	ImageNet	**0.8811** ± **0.0334**	**0.9006** ± **0.0218**	**0.8811** ± **0.0334**	**0.8873** ± **0.0290**
	RadiologyNET	0.8383 ± 0.0224	0.8406 ± 0.0182	0.8383 ± 0.0224	0.8380 ± 0.0204
	RadImageNet	0.3926 ± 0.2439	0.3861 ± 0.2785	0.3926 ± 0.2439	0.3284 ± 0.2771
DenseNet121	Baseline	0.7789 ± 0.0602	0.8218 ± 0.0503	0.7789 ± 0.0602	0.7873 ± 0.0583
	ImageNet	0.8473 ± 0.0174	0.8878 ± 0.0126	0.8473 ± 0.0174	0.8609 ± 0.0154
	RadiologyNET	**0.9101** ± **0.0298**	**0.9213** ± **0.0224**	**0.9101** ± **0.0298**	**0.9127** ± **0.0257**
	RadImageNet	0.2903 ± 0.0643	0.2242 ± 0.0919	0.2903 ± 0.0643	0.1700 ± 0.0622
EfficientNet-B3	Baseline	0.3318 ± 0.0305	0.3795 ± 0.0376	0.3318 ± 0.0305	0.2963 ± 0.0404
	ImageNet	0.9033 ± 0.0100	0.9107 ± 0.0115	0.9033 ± 0.0100	0.9059 ± 0.0097
	RadiologyNET	**0.9199** ± **0.0079**	**0.9246** ± **0.0066**	**0.9199** ± **0.0079**	**0.9190** ± **0.0070**
MobileNetV3-Large	Baseline	0.2500 ± 0.0000	0.0834 ± 0.0060	0.2500 ± 0.0000	0.1250 ± 0.0071
	ImageNet	0.7005 ± 0.1594	0.7726 ± 0.0655	0.7005 ± 0.1594	0.6649 ± 0.1777
	RadiologyNET	**0.8471** ± **0.0367**	**0.8723** ± **0.0316**	**0.8471** ± **0.0367**	**0.8548** ± **0.0332**

**Table 3:** Results on the BRISC brain tumour MRI dataset, reported as mean ± standard deviation across ten runs. The highest value per metric within each architecture is shown in bold.

Model	Weight	Accuracy	Precision	Recall	F1
ResNet50	Baseline	0.9203 ± 0.0397	0.9300 ± 0.0347	0.9203 ± 0.0397	0.9217 ± 0.0412
	ImageNet	**0.9885** ± **0.0040**	**0.9891** ± **0.0039**	**0.9885** ± **0.0040**	**0.9888** ± **0.0039**
	RadiologyNET	0.9830 ± 0.0036	0.9843 ± 0.0031	0.9830 ± 0.0036	0.9836 ± 0.0034
	RadImageNet	0.9730 ± 0.0035	0.9748 ± 0.0037	0.9730 ± 0.0035	0.9738 ± 0.0035
InceptionV3	Baseline	0.9331 ± 0.0147	0.9361 ± 0.0135	0.9331 ± 0.0147	0.9336 ± 0.0143
	ImageNet	**0.9918** ± **0.0018**	**0.9920** ± **0.0019**	**0.9918** ± **0.0018**	**0.9919** ± **0.0018**
	RadiologyNET	0.9721 ± 0.0040	0.9742 ± 0.0036	0.9721 ± 0.0040	0.9729 ± 0.0039
	RadImageNet	0.9550 ± 0.0041	0.9580 ± 0.0044	0.9550 ± 0.0041	0.9562 ± 0.0042
DenseNet121	Baseline	0.9722 ± 0.0060	0.9732 ± 0.0068	0.9722 ± 0.0060	0.9725 ± 0.0064
	ImageNet	**0.9894** ± **0.0024**	**0.9899** ± **0.0024**	**0.9894** ± **0.0024**	**0.9896** ± **0.0024**
	RadiologyNET	0.9856 ± 0.0016	0.9865 ± 0.0017	0.9856 ± 0.0016	0.9860 ± 0.0016
	RadImageNet	0.3626 ± 0.1879	0.2703 ± 0.2237	0.3626 ± 0.1879	0.2597 ± 0.2264
EfficientNet-B3	Baseline	0.8804 ± 0.0184	0.8846 ± 0.0167	0.8804 ± 0.0184	0.8809 ± 0.0189
	ImageNet	**0.9888 ± 0.0033**	**0.9891 ± 0.0031**	**0.9888 ± 0.0033**	**0.9889 ± 0.0032**
	RadiologyNET	0.9862 ± 0.0026	0.9871 ± 0.0028	0.9862 ± 0.0026	0.9866 ± 0.0027
MobileNetV3-Large	Baseline	0.8108 ± 0.0385	0.8222 ± 0.0271	0.8108 ± 0.0385	0.8054 ± 0.0458
	ImageNet	**0.9855** ± **0.0028**	**0.9860** ± **0.0028**	**0.9855** ± **0.0028**	**0.9857** ± **0.0028**
	RadiologyNET	0.9776 ± 0.0029	0.9787 ± 0.0028	0.9776 ± 0.0029	0.9781 ± 0.0029

**Table 4:** Results on the FracAtlas dataset, reported as mean ± standard deviation across ten runs. The highest value per metric within each architecture is shown in bold.

Model	Weight	Accuracy	Precision	Recall	F1
ResNet50	Baseline	0.8389 ± 0.0072	0.8644 ± 0.0099	0.9549 ± 0.0167	0.9072 ± 0.0046
	ImageNet	**0.8904** ± **0.0140**	**0.9237** ± **0.0091**	0.9455 ± 0.0238	**0.9342** ± **0.0094**
	RadiologyNET	0.8657 ± 0.0082	0.8739 ± 0.0100	**0.9786** ± **0.0105**	0.9232 ± 0.0045
	RadImageNet	0.8693 ± 0.0048	0.8935 ± 0.0078	0.9556 ± 0.0110	0.9234 ± 0.0030
InceptionV3	Baseline	0.8374 ± 0.0085	0.8792 ± 0.0161	0.9319 ± 0.0298	0.9042 ± 0.0070
	ImageNet	**0.8892** ± **0.0094**	**0.9102** ± **0.0163**	**0.9612** ± **0.0203**	**0.9347** ± **0.0057**
	RadiologyNET	0.8451 ± 0.0093	0.8883 ± 0.0068	0.9293 ± 0.0179	0.9082 ± 0.0065
	RadImageNet	0.8480 ± 0.0366	0.8863 ± 0.0202	0.9360 ± 0.0304	0.9103 ± 0.0225
DenseNet121	Baseline	0.8554 ± 0.0040	0.8826 ± 0.0122	0.9519 ± 0.0198	0.9157 ± 0.0034
	ImageNet	**0.9038** ± **0.0097**	**0.9237** ± **0.0133**	**0.9634** ± **0.0178**	**0.9429** ± **0.0059**
	RadiologyNET	0.8685 ± 0.0114	0.8905 ± 0.0114	0.9588 ± 0.0179	0.9232 ± 0.0071
	RadImageNet	0.7962 ± 0.0838	0.8673 ± 0.0162	0.8917 ± 0.1279	0.8729 ± 0.0716
EfficientNet-B3	Baseline	0.8389 ± 0.0054	0.8542 ± 0.0079	0.9707 ± 0.0129	0.9086 ± 0.0033
	ImageNet	**0.9095** ± **0.0092**	**0.9207** ± **0.0107**	**0.9745** ± **0.0100**	**0.9467** ± **0.0053**
	RadiologyNET	0.8745 ± 0.0063	0.8946 ± 0.0060	0.9612 ± 0.0073	0.9267 ± 0.0037
MobileNetV3-Large	Baseline	0.8098 ± 0.0268	0.8544 ± 0.0070	0.9279 ± 0.0418	0.8891 ± 0.0185
	ImageNet	**0.8779** ± **0.0094**	**0.9139** ± **0.0178**	**0.9416** ± **0.0194**	**0.9272** ± **0.0054**
	RadiologyNET	0.8538 ± 0.0098	0.8957 ± 0.0038	0.9313 ± 0.0139	0.9131 ± 0.0064

**Table 5:** Friedman test statistics and post hoc Wilcoxon signed-rank test results (Holm-corrected *p*-values) for pairwise comparisons between initialisation strategies, by architecture and dataset (n=10 runs per configuration). The value in parentheses next to $\chi^{2}$ denotes the degrees of freedom, given by the number of initialisation strategies compared minus one. B = Baseline, I = ImageNet, R = RadiologyNET, RI = RadImageNet. Cells marked “–” indicate the comparison was not applicable, as RadImageNet weights were unavailable for EfficientNet-B3 and MobileNetV3-Large. Values marked with ** indicate statistical significance at α=0.05.

	Friedman		Wilcoxon (Holm-Corrected *p*)					
Architecture	$\chi^{2}$ (df)	*p*	B–I	B–R	B–RI	I–R	I–RI	R–RI
Chest CT scan dataset								
ResNet50	23.76 (3)	<0.0001 **	0.0117 **	0.0117 **	0.0117 **	0.0117 **	0.0117 **	1.0000
InceptionV3	21.72 (3)	<0.0001 **	0.0117 **	0.0840	0.0293 **	0.0117 **	0.0117 **	0.0293 **
DenseNet121	28.92 (3)	<0.0001 **	0.0195 **	0.0117 **	0.0117 **	0.0117 **	0.0117 **	0.0117 **
EfficientNet-B3	18.20 (2)	0.0001 **	0.0059 **	0.0059 **	–	0.0059 **	–	–
MobileNetV3-Large	18.20 (2)	0.0001 **	0.0059 **	0.0059 **	–	0.0059 **	–	–
BRISC brain tumour MRI dataset								
ResNet50	28.08 (3)	<0.0001 **	0.0117 **	0.0117 **	0.0117 **	0.0273 **	0.0117 **	0.0117 **
InceptionV3	28.92 (3)	<0.0001 **	0.0117 **	0.0117 **	0.0117 **	0.0117 **	0.0117 **	0.0117 **
DenseNet121	28.08 (3)	<0.0001 **	0.0117 **	0.0117 **	0.0117 **	0.0117 **	0.0117 **	0.0117 **
EfficientNet-B3	16.80 (2)	0.0002 **	0.0059 **	0.0059 **	–	0.1309	–	–
MobileNetV3-Large	20.00 (2)	<0.0001 **	0.0059 **	0.0059 **	–	0.0059 **	–	–
FracAtlas dataset								
ResNet50	21.36 (3)	<0.0001 **	0.0117 **	0.0117 **	0.0117 **	0.0547	0.0352 **	0.7695
InceptionV3	21.72 (3)	<0.0001 **	0.0117 **	0.3164	0.3164	0.0117 **	0.0117 **	0.3164
DenseNet121	26.76 (3)	<0.0001 **	0.0117 **	0.0371 **	0.0273 **	0.0117 **	0.0117 **	0.0117 **
EfficientNet-B3	20.00 (2)	<0.0001 **	0.0059 **	0.0059 **	–	0.0059 **	–	–
MobileNetV3-Large	18.20 (2)	0.0001 **	0.0059 **	0.0059 **	–	0.0059 **	–	–

## Data Availability

The data used in the study are openly available at https://www.kaggle.com/datasets/mohamedhanyyy/chest-ctscan-images (accessed on 20 July 2026) (Chest CT), https://doi.org/10.1038/s41597-026-06753-y (accessed on 18 September 2026) (BRISC), and https://doi.org/10.1038/s41597-023-02432-4 (accessed on 15 September 2026) (FracAtlas). The pretrained weights are available in GitHub at https://github.com/BMEII-AI/RadImageNet (accessed on 20 July 2026) (RadImageNet) and https://github.com/AIlab-RITEH/RadiologyNET-TL-models (accessed on 20 July 2026) (RadiologyNET). Data generated during the experiments are available from the corresponding author upon request.

## Abbreviations

The following abbreviations are used in this manuscript:

BRISC	Brain Tumor Image Segmentation and Classification
CR	Computed Radiography
CT	Computed Tomography
MRI	Magnetic Resonance Imaging
NSCLC	Non-small Cell Lung Cancer
RF	Radiofluoroscopy
t-SNE	t-distributed Stochastic Neighbour Embedding
US	Ultrasound
XA	X-ray Angiography

## Appendix A. Implementation Details and Training Time

All experiments were conducted on a Gigabyte G492-Z50 HPC GPU server equipped with two AMD EPYC 7702 64-core processors, 1 TB of DDR4 RAM, and four NVIDIA L40S GPUs with 48 GB of memory each. Each model was trained on a single GPU. Experiments were implemented in PyTorch 2.13.0 (CUDA 13.0), torchvision 0.28.0, torchmetrics 1.8.2, and scikit-learn 1.9.0 (t-SNE). The *i*-th run of every configuration used the seed 42+i (seeds 43–52) for all random number generators, with deterministic cuDNN algorithms enabled. Table A1 reports the average training time per run for each combination of architecture, initialisation strategy, and dataset.

**Table A1:** Average training time per run (in seconds), reported as mean ± standard deviation across ten runs, by architecture, dataset, and weight-initialisation strategy. The Overall column pools across all available weight-initialisation strategies for the corresponding architecture and dataset. Cells marked “–” indicate the comparison was not applicable.

Model	Baseline	ImageNet	RadiologyNET	RadImageNet	Overall
Chest CT scan dataset					
ResNet50	96.0 s ± 32.2 s	70.4 s ± 8.0 s	134.8 s ± 28.8 s	266.4 s ± 36.0 s	141.9 s ± 80.6 s
InceptionV3	132.0 s ± 28.7 s	83.9 s ± 21.2 s	286.2 s ± 28.1 s	118.4 s ± 75.4 s	155.1 s ± 89.3 s
DenseNet121	165.4 s ± 31.9 s	120.8 s ± 21.3 s	227.8 s ± 58.6 s	96.9 s ± 1.0 s	152.7 s ± 60.9 s
EfficientNet-B3	80.2 s ± 27.7 s	118.0 s ± 22.7 s	111.1 s ± 27.2 s	–	103.1 s ± 30.7 s
MobileNetV3-Large	38.1 s ± 4.4 s	69.6 s ± 30.9 s	74.9 s ± 18.9 s	–	60.9 s ± 26.6 s
BRISC brain tumour MRI dataset					
ResNet50	907.8 s ± 245.4 s	561.5 s ± 150.6 s	563.9 s ± 325.4 s	320.1 s ± 57.0 s	588.3 s ± 303.0 s
InceptionV3	639.0 s ± 123.7 s	755.3 s ± 161.1 s	1033.8 s ± 120.8 s	835.0 s ± 174.2 s	815.8 s ± 205.6 s
DenseNet121	1069.9 s ± 178.7 s	1058.2 s ± 226.6 s	1076.0 s ± 174.3 s	549.3 s ± 95.2 s	938.4 s ± 284.9 s
EfficientNet-B3	704.7 s ± 192.3 s	751.9 s ± 204.6 s	551.0 s ± 118.3 s	–	669.2 s ± 195.7 s
MobileNetV3-Large	323.7 s ± 58.6 s	418.7 s ± 117.2 s	278.6 s ± 31.8 s	–	340.3 s ± 97.3 s
FracAtlas dataset					
ResNet50	404.0 s ± 161.0 s	183.6 s ± 0.6 s	183.7 s ± 0.6 s	191.4 s ± 8.5 s	240.7 s ± 124.1 s
InceptionV3	606.1 s ± 149.8 s	473.8 s ± 2.6 s	505.4 s ± 42.0 s	557.4 s ± 82.5 s	535.7 s ± 101.5 s
DenseNet121	755.8 s ± 110.8 s	648.6 s ± 2.3 s	647.5 s ± 3.9 s	615.6 s ± 100.0 s	666.9 s ± 91.6 s
EfficientNet-B3	435.9 s ± 28.5 s	419.5 s ± 1.2 s	414.9 s ± 3.5 s	–	423.4 s ± 18.9 s
MobileNetV3-Large	337.0 s ± 18.9 s	287.6 s ± 2.0 s	287.4 s ± 1.4 s	–	304.0 s ± 25.8 s

## Appendix B. t-SNE Visualisations

Figure A1, Figure A2 and Figure A3 show t-SNE projections of test-set feature embeddings extracted from the penultimate layer of ResNet50 for the Chest CT scan, BRISC, and FracAtlas datasets, respectively. ResNet50 was selected as the only architecture that trained stably under all four initialisation strategies, allowing for a direct comparison of the learned representations. The projections were computed with scikit-learn using two output dimensions, a perplexity of 15, and a random state of 42, with all other parameters left at their default values.

The ResNet50 t-SNE projections in Figure A1 show visible differences in the organisation of the learned feature spaces. In the Baseline representation (Figure A1a), normal samples form a separate group (although not entirely homogeneous), whereas the three carcinoma subtypes overlap substantially. The pretrained configurations (Figure A1b–d) instead form distinct, class-specific clusters for each carcinoma subtype. In all three pretrained configurations, however, the normal cluster also contains a number of carcinoma samples, predominantly adenocarcinoma, which is consistent with the corresponding confusion matrices (Figure 3).

The ResNet50 t-SNE projections in Figure A2 show four well-defined clusters corresponding to the four classes for all initialisation strategies, with *no tumour* samples forming a clearly isolated group in every case. In the Baseline representation (Figure A2a), the glioma and meningioma clusters lie adjacent to each other with visible mixing at their boundary, and several samples from both classes are located within the pituitary cluster. The ImageNet and RadiologyNET representations (Figure A2b,c) form compact, well-separated clusters, with only a small number of samples bridging the glioma and meningioma groups. The RadImageNet representation (Figure A2d) shows a similar structure, but with a wider contact region between glioma and meningioma.

The ResNet50 t-SNE projections (Figure A3) for the FracAtlas dataset reflect the class imbalance of the test set, with fractured samples forming a small minority. In the Baseline representation (Figure A3a), fractured samples are scattered throughout the projection and intermixed with non-fractured samples, without forming a distinct group. The ImageNet representation (Figure A3b) shows the clearest separation, with most fractured samples forming a compact cluster at the edge of the projection. In the RadiologyNET and RadImageNet representations (Figure A3c,d), fractured samples are concentrated in one region of the projection but considerable overlap with non-fractured samples is still present.
